# IGFBP‐5 Potentially Mediates the Association of Brain Insulin Signaling with Late‐Life Cognitive Decline

**DOI:** 10.1002/alz.087777

**Published:** 2025-01-03

**Authors:** Han Tong, Ana W. Capuano, David A. Bennett, Rexford S. Ahima, Steven E. Arnold, Zoe Arvanitakis

**Affiliations:** ^1^ Rush University Medical Center, Chicago, IL USA; ^2^ Johns Hopkins University School of Medicine, Baltimore, MD USA; ^3^ Massachusetts General Hospital, Harvard Medical School, Boston, MA USA

## Abstract

**Background:**

Abnormal brain insulin signaling has been associated with Alzheimer’s disease pathology and a faster rate of late‐life cognitive decline. However, the underlying mechanisms remain unclear. In this study, we examined whether AD‐related cortical proteins identified using targeted‐proteomics play a role in the association of brain insulin signaling and cognitive decline.

**Method:**

Among the Religious Orders Study participants who underwent annual clinical and comprehensive neuropsychological testing (summarized as global and domain‐specific cognitive measures), 118 deceased and autopsied older individuals (59 with diabetes matched to 59 without, by age at death, sex, and education) had both brain (prefrontal cortex) insulin signaling (by ELISA and immunohistochemistry, including RAC‐alpha serine/threonine‐protein kinase or AKT1) and AD‐related cortical protein measurements. Levels of five cortical proteins including insulin‐like growth factor‐binding protein‐5 (IGFBP‐5), inositol‐tetrakisphosphate 1‐kinase (ITPK1), heat shock protein B2 (HSPB2), adenylate kinase 4 (AK4), and plexin B1(PLXNB1) were measured using selection reaction monitoring quantitative proteomics. We conducted linear mixed‐effect model analyses adjusted for age at death, sex, and education to examine associations of cortical proteins and insulin signaling measures with longitudinally assessed cognitive function.

**Result:**

A higher level of IGFBP‐5 (estimate = 0.260, SE = 0.092, p = 0.005) and a lower level of ITPK1 (estimate = ‐0.278, SE = 0.092, p = 0.003) were each associated with a higher level of pT^308^AKT1 /total AKT1. In addition, a higher level of pT^308^AKT1 /total AKT1 was associated with a faster rate of decline in global cognition (estimate = ‐0.035, SE = 0.013, p = 0.007), and most cognitive domains including episodic memory (estimate = ‐0.039, SE = 0.014, p = 0.005), working memory (estimate = ‐0.031, SE = 0.009, p = 0.001), perceptual speed (estimate = ‐0.026, SE = 0.011, p = 0.015), and visuospatial abilities (estimate = ‐0.017, SE = 0.007, p = 0.019). Yet, when the level of IGFBP‐5 and its interaction with time were added to the models as covariates, the level of pT^308^AKT1 /total AKT1 was no longer associated with the decline rate of global cognition (estimate = ‐0.023, SE = 0.012, p = 0.067) and cognitive domains including perceptual speed (estimate = ‐0.018, SE = 0.010, p = 0.082) and visual spatial abilities (estimate = ‐0.014, SE = 0.007, p = 0.052).

**Conclusion:**

AD‐related cortical proteins IGFBP‐5 and ITPK1 are each associated with insulin signaling AKT1 phosphorylation in postmortem human brain. Further, IGFBP‐5 potentially mediates the association of AKT1 phosphorylation with late‐life cognitive decline.

